# Optimizing pterygoid implant placement without sinus intrusion in edentulous vietnamese patients: A comprehensive tomographic analysis and cross-sectional study

**DOI:** 10.4317/jced.61787

**Published:** 2024-11-01

**Authors:** Dau Cao Luong, Le Duc Lanh, Vo Lam Thuy, Pham Thi Huong Loan

**Affiliations:** 1 Department of Oral Implantology, Faculty of Odonto-Stomatology, University of Medicine and Pharmacy at Ho Chi Minh City; 2 Faculty of Odonto-Stomatology, Hong Bang University

## Abstract

**Background:**

Severe maxillary resorption presents challenges in dental implant placement. This research aims to assess the feasibility, angular orientation, and appropriate length of pterygoid implants in patients with significant maxillary atrophy.

**Material and Methods:**

The study examined Cone Beam Computed Tomography (CBCT) scans from 60 completely edentulous patients classified as Cawood and Howell’s Classes V or VI, with less than 4mm residual bone height in their posterior maxilla. Experienced oral and maxillofacial surgeons and researchers conducted virtual pterygoid implant placement, evaluating various implant positions.

**Results:**

Position C was the most frequent, comprising 34.6% of cases evaluated. The average antero-posterior angle across all positions was 51.82±5.57 degrees, and the bucco-lingual angle was 74.15±16.53 degrees relative to the Frankfort horizontal plane. The optimal location for implant neck placement was approximately 10 mm from the most distal point of maxillary tuberosity, angled 50 degrees antero-posteriorly and 75 degrees bucco-lingually. While 18 mm implants were typically used, lengths of 20-22 mm were sometimes necessary for bicortical anchorage.

**Conclusions:**

This study demonstrates the viability of pterygoid implants even in cases of significant maxillary atrophy. The findings emphasize the importance of adapting implant placement strategies to individual patient anatomies. Further research may be needed to refine techniques for patients with severe maxillary resorption.

** Key words:**Pterygoid Implant, Edentulous Patient, CBCT (Cone Beam Computed Tomography), Tomographic analysis.

## Introduction

Immediate full-arch implant-supported fixed prostheses can significantly enhance the quality of life for entirely edentulous individuals, providing full dental functionality again (1). However, treating patients suffering from moderate to severe maxillary resorption presents significant clinical challenges. These challenges stem from reduced bone volume and quality due to alveolar bone resorption, sinus pneumatization, and the inherent paucity of density in the maxillary region. One method to address these challenges effectively involves placing implants strategically within areas of the jaw where bone integrity remains relatively robust after tooth loss. Placement sites like zygomatic and pterygoid bones are particularly advantageous for implant placement as they offer a reliable foundation in situations with limited bone volume or quality (2-5).

Dr. Tulasne first introduced pterygoid implants into dentistry in 1989 and they have become an important milestone in implantology, especially for patients experiencing significant bone loss in the posterior maxilla where traditional methods often fall short (6). Recent evidence demonstrates the success of using rough-surfaced pterygoid implants at an astounding success rate of 95.5%, comparable to conventional implants deployed in both mandibular and maxillary regions (7). Although pterygoid implants may be effective, their application requires a careful and meticulous approach due to their potential risk of severe complications (8-10). Acquiring expertise in applying them requires physicians possessing thorough knowledge of anatomy of the pterygoid region along with the necessary level of surgical experience (11).

Implant dentistry remains in pursuit of a standard technique for placing pterygoid implants with no clear consensus in this field (11-14). An array of recommended techniques has been presented throughout the literature. Certain studies advocate positioning implants at 45 degrees anterior-posteriorly relative to the Frankfort horizontal plane (12,14). In comparison, alternative studies propose placing the implant along a bone corridor at 70 degrees to enhance biomechanical stability and ensure long-term success (13). Xavier Rodriguez performed an anatomical study utilizing cone-beam computed tomography (CBCT) and determined that for optimal implant placement on pterygoid patients using this technology, the optimal angle ranged between 72.5° to 74.19 , with an approximately 22.5mm bone column length (15,16). On the contrary, Uchida conducted his 2017 analysis on the Japanese cohort and revealed broader variation ranging from 52.3° to 75.1°, depending on implant neck location from the first molar (17). Zhang’s 2023 proposition suggested placing the pterygoid implant neck 12.91mm from the most posterior point of maxillary tuberosity and deviating 45.08o from the Frankfort horizontal plane for maximum oral hygiene benefits (18). Sun (2023) recommended deviating 45 degrees from the Frankfort horizontal plane for placing pterygoid implants and positioning their neck 10 mm away from the pterygomaxillary joint according to prosthesis guide recommendations. With this approach, up to 91.6% of virtual pterygoid implants intrude into maxillary sinus (19). Unfortunately, their study samples included partially and fully edentulous patients; no studies specifically explored optimal placement and angle for placing implants for fully edentulous patients.

This study’s purpose was to analyze and compare the feasibility, angular orientations and lengths of pterygoid implants positioned at the midpoint of the maxillary ridge for maxillary teeth with increments of 6, 8, 10 12 14mm from tuberosity’s most posterior point.

## Material and Methods

-Study Protocol 

Local general hospital’s Institutional Review Board (IRB-VN02018) approved this study’s ethical approval, guaranteeing strict adherence to ethical guidelines. Patient data was processed through extensive encoding procedures designed to protect confidentiality and privacy. This was a cross-sectional study which Cone Beam Computed Tomography (CBCT) scans from fully edentulous patients requiring dental implant treatment were obtained from local general hospital imaging department from January 2022 until December 2023 for this investigation - an initiative between the research team and imaging department ensured reliable data acquisition processes both parties partnered closely in this data acquisition process.

Participant selection criteria have been carefully designed to include fully edentulous patients from Cawood and Howell class V or VI with a remaining bone height less than 4 mm in their maxillary molar region (20). Conversely, exclusion criteria outline parameters such as age (under 18 years old), impacted teeth or roots in the maxillary molar region, unclear radiographs due to motion artifacts, history of traffic accidents, or genetic maxillary bone defects as exclusionary criteria - thus assuring reliability within participant cohort and mitigating potential confounding variables.

The CBCT imaging was conducted using the Plamenca ProMax® 3D Mid CBCT scanner (Helsinki, Finland), employing the following scanning parameters: 8 mA, 90 kV, with a voxel size of 0.2 mm and a field of view (FOV) measuring 200 mm × 100 mm. The scan duration was 18 seconds. To ensure standardization and consistency, the CBCT images were oriented relative to two reference planes: the Frankfort horizontal plane (for sagittal view) and the pupilar plane (for frontal view), both aligned parallel to the floor. Patient CBCT data meeting the predetermined eligibility criteria were obtained in DICOM format. Subsequently, these DICOM files were imported into DTX Studio™ planning software for comprehensive tomographic analysis.

-Anatomical and Radiological Measurements

**Figure 1:**
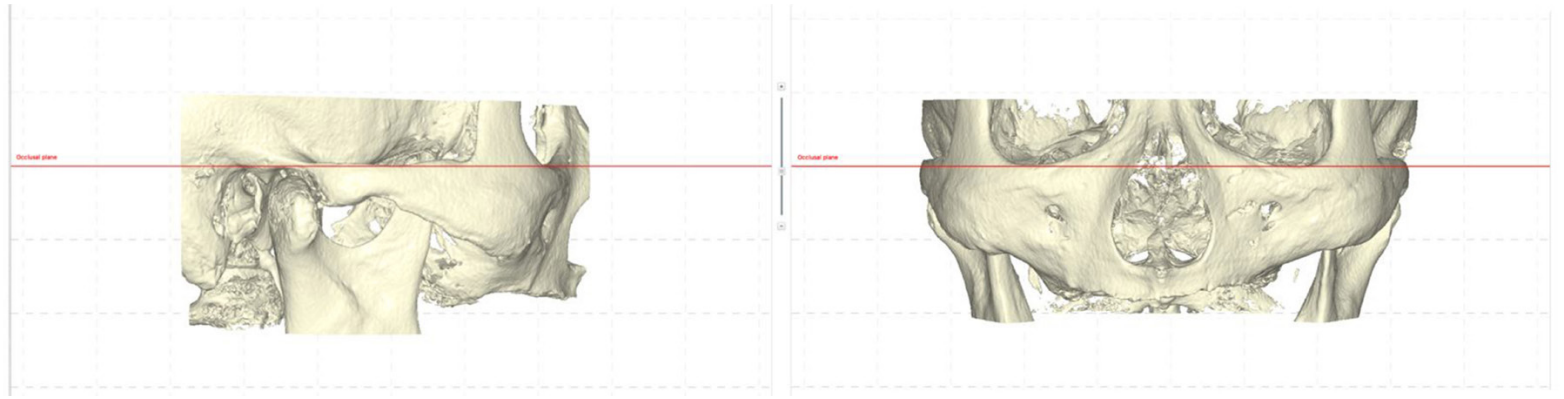
Upper: The Frankfort horizontal plane drawn with DTX Studio™ Software. Lower: The bilateral, greater palatine nerve canals and panoramic curve are drawn on the software (DTX Studio™).

In this study, the Frankfort horizontal plane was used as the standard reference line for orientation, as visualized in Figure 1. Utilizing DTX Studio™ software, the greater palatine canals were precisely identified and annotated on both sides to facilitate accurate anatomical mapping. A panoramic curve, detailed in Figure 1, was constructed from the maxillary tuberosity through the midpoint of the maxillary ridge anteriorly, intersecting the pterygomaxillary junction’s most concave point at the posterior nasal spine level, providing an extensive anatomical perspective.

**Figure 2:**
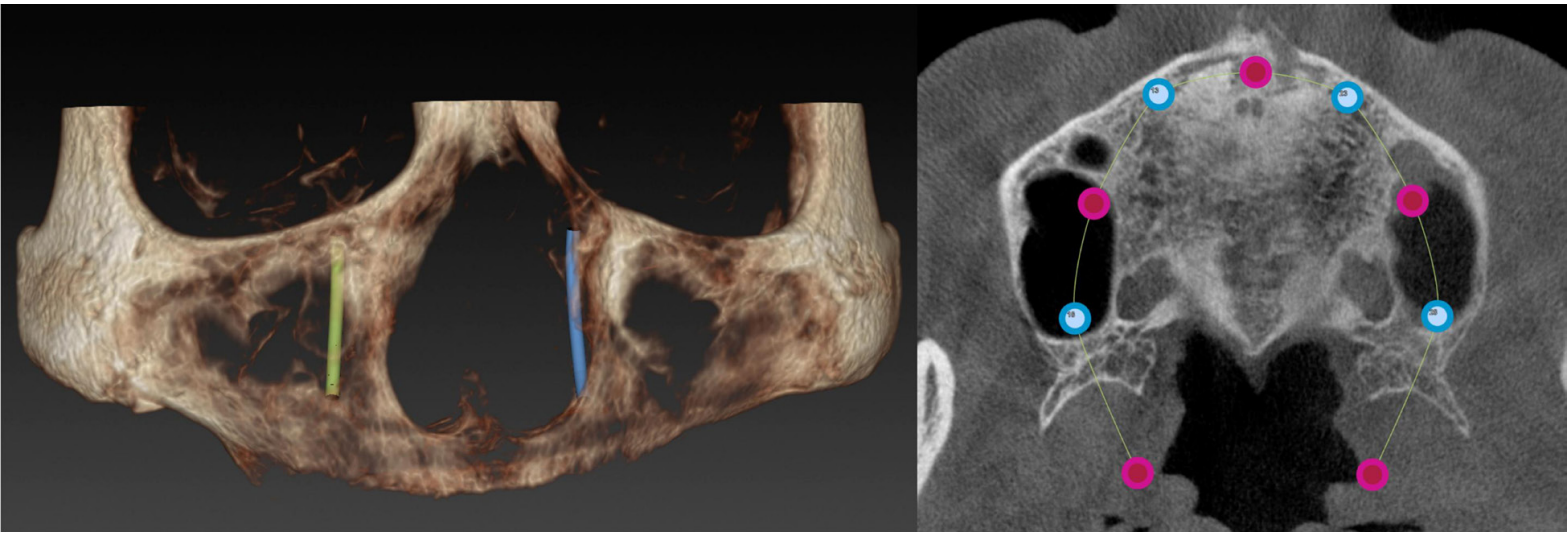
Sagittal View Diagram Illustrating Key Landmarks.

Point M was defined as the horizontal projection of the tuberosity’s posterior end, in alignment with the maxillary ridge’s horizontal plane. Subsequently, Points A to E were located along this central axis at intervals of 6, 8, 10, 12, and 14 mm from Point M, as shown in Figure 2, for the analysis of implant placement.

The minimum residual bone height at the molars sites (RBH), and width of the maxillary ridge are measured at points A, B, C, D and E (Fig. 2).

-Virtual Implant Placement

**Figure 3:**
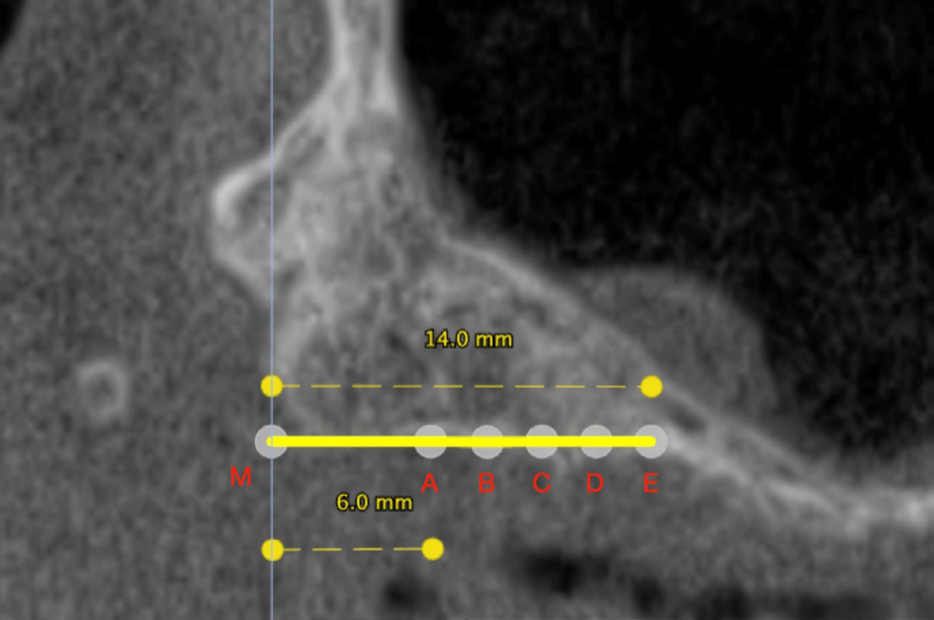
Upper: Residual Bone Height at molars site and Bone Dimensions at Points C. Lower: The design and measurement of the virtual pterygoid implant’s parameters were conducted using DTX Studio™ software.

Two independent researchers took charge of the tomographic measurements and implant placement, while a highly skilled oral and maxillofacial surgeon and researcher with over 30 years of expertise supervised the procedure, (Fig. 3).

The virtual pterygoid implants (Nobel Speedy® Groovy), measuring between 10 mm to 25 mm in length with a uniform diameter of 4.0 mm, are meticulously placed using entry points at 6mm (Point A), 8mm (Point B), 10 mm (Point C), 12 mm (Point D) and 14mm (Point E), from the most posterior point of tuberosity known as Point M. This ensures placement of the implant neck in the centre of the maxillary ridge as well as precise placement of its apex at the most concave point of the pterygoid process. Bicortical anchorage is the key to achieving maximum stability with minimal penetration to the pterygoid fossa. To be considered a successful implant placement, specific criteria must be fulfilled: the implant must be surrounded in bone, placed 2 mm away from the greater palatine canal, and avoid intrusion into the sinus cavity.

The following parameters were measured: 1) possibility to place implants without sinus intrusion at various positions; 2) angle of the implant relative to the Frankfort horizontal plane in the anterior-posterior and buccal-lingual directions at various positions; 3) distribution of pterygoid implants length used at various positions; 4) optimal position, angulation, and length of pterygoid implants.

-Statistical analysis

Data was analyzed with SPSS (15.0, SPSS Inc., Chicago, IL, USA) using a P-value < 0.05 for statistical significance. To assess intra-observer reliability and error analysis, measurements on all CBCT images were repeated by each observer one week after initial measurements, providing for determination of intra-observer error analysis.

Data was presented as mean ± standard deviation. Normality was assessed using the Kolmogorov-Smirnov test; Paired T-tests were utilized to compare left and right variables; Gender T-tests were employed to examine measurement differences between men and women age groups; Chi-square test and one-way ANOVA with LSD and Bonferroni post-hoc was then conducted to compare implant placement probability, length, angle deviation from Frankfort Plane at A, B, C D and E locations.

## Results

-Residual bone height and width

A total of 60 Vietnamese patients (26 males and 34 females) with severely atrophied edentulous maxillary ridges were eligible for inclusion in this study. Their mean age was 63.23 ± 6.8 years (range 43–84 years). The minimum residual height of the maxillary ridge on the right and left sides was 2.11 ± 1.06 mm and 2.02 ± 1.07 mm, respectively, with no statistically significant difference between the minimum residual bone heights on the right and left sides.

The height of the bone showed a gradual decrease from position A (8.02±0.31mm) to position E (3.99±0.19 mm), with a statistically significant difference observed (Supplement 1). Additionally, bone width was larger anteriorly at positions C, D, and E compared to the posterior positions of the tuberosity (A, B).

-Possibility to place implants without sinus intrusion at various positions 

The data showed no statistically significant difference in the possibility of placing implants without involving the maxillary sinus between the right and left sides (*p* > 0.05). The success rates for avoiding sinus intrusion during implant placement are comparable on both sides. However, there was a noticeable anterior-posterior gradient in success rates. It became increasingly difficult to place implants without sinus intrusion as one moves from position A to position E. On the right side, the success rate decreased from 90% at position A to 50% at position E. On the left side, the success rate dropped from 83.33% at position A to 36.7% at position E. Notably, the transition from position A to position B did not show a statistically significant reduction in implant placement success (*p* > 0.05) ( Table 1).

-Angle of the implant relative to the frankfort horizontal plane in the anterior-posterior and buccal-lingual directions at various positions

In the anteroposterior axis (sagittal view), a significant variation was observed in the mean angulation of the implant relative to the Frankfort horizontal plane, ranging from position A (68±6.9 degrees) to position E (32.7±8.5 degrees). Similarly, in the buccolingual axis (frontal view), a notable difference in mean angulation relative to the Frankfort horizontal plane was evident, with values shifting from position A (78.2 ± 5.4 degrees) to position E (67.6 ± 10.8 degrees). This analysis highlighted a substantial reduction in the deviation from the Frankfort plane, both in anterior-posterior and buccal-lingual directions, when the angulation of the implant neck was adjusted from a posterior to an anterior position, ( Table 2).

-Distribution of pterygoid implants length used at various positions

The required implant length gradually increased from position A to position E. In the more posterior positions, specifically A and B, there was a clear preference for shorter implants, with lengths ranging between 10 and 13 mm. In contrast to positions A and B, there was an increase in the use of longer implants, such as 18 mm and 20 - 22 mm, as one moves towards positions D and E.

**Figure 4:**
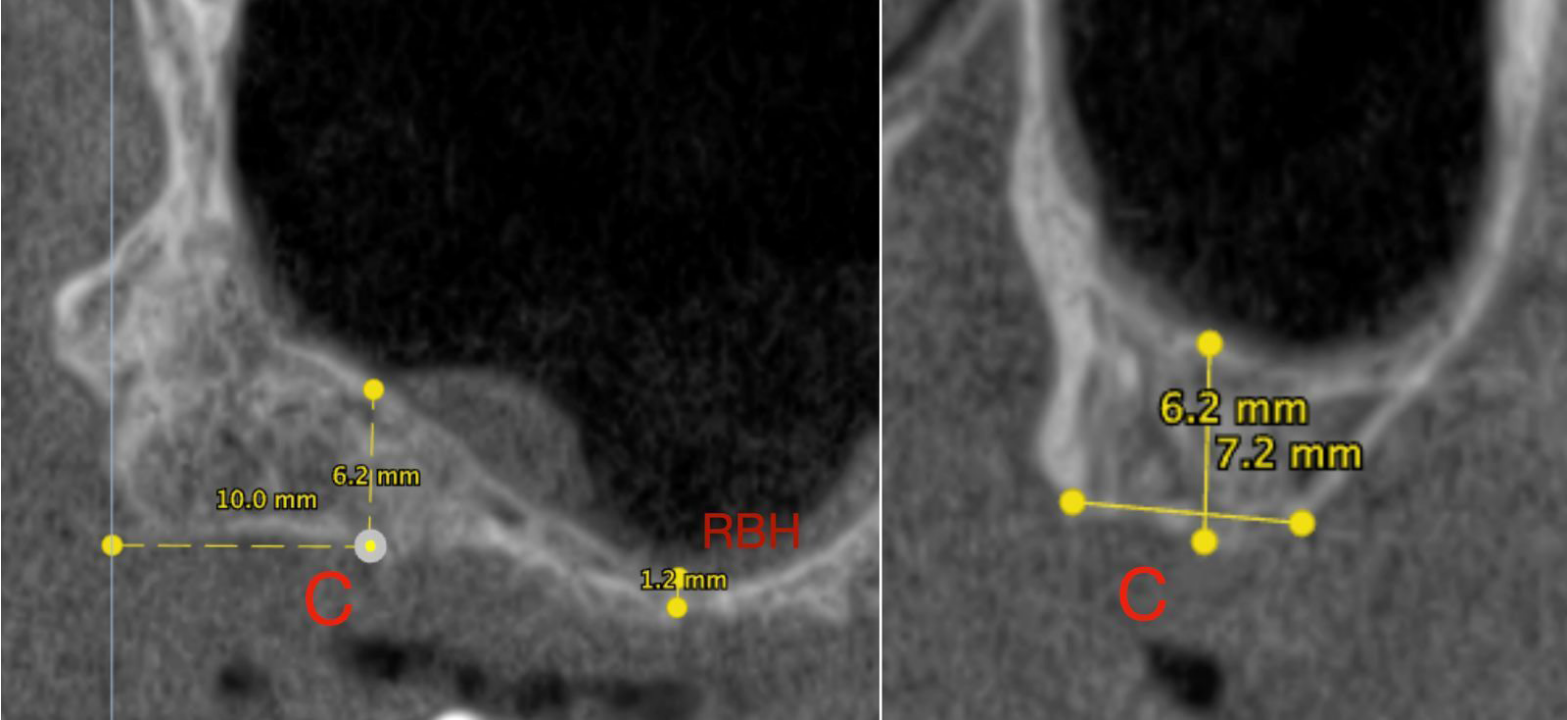
Distribution of pterygoid implants length used at various positions.

The widespread utilization of 18mm implants in intermediate positions B, C, and D underlined this length as a versatile option. It effectively bridged the divide between the shorter implants preferred in posterior areas and the longer implants preferred in anterior areas, as illustrated in Figure 4, ( Table 3).

-Optimal position, angulation, and length of pterygoid implants 

The distribution of implant positions from A through E revealed a preference for position C (34.6%), suggesting it as the most favored or clinically significant location for pterygoid implants, followed by position B (26%), D (21.2 %), A (10.6%), and E (7.7%). The antero-posterior angle showed a gradual decrease from position A (58±8.3 degrees) to position E (48.81±2.61 degrees), with an overall average angle of approximately 51.82±5.57 degrees across all positions.

In terms of the buccal-lingual angle, there was noticeable variability, with the highest angle observed in position A (76.86±5.27 degrees) and the lowest in position E (68.68±4.5 degrees). The overall average angle was 74.15±16.53 degrees. Regarding implant length, there was a clear progression from shorter implants in position A (13mm, range 11.5-15) to longer implants in position E (21mm, range 20-22), with 18mm (range 15-18) being the most commonly recommended length across positions, ( Table 4).

## Discussion

In this study, all patients presented with complete edentulism, exhibiting severe maxillary bone resorption classified as Cawood and Howell’s Types V and VI (20). As the study progressed anteriorly from posterior regions, the height of the maxillary bone decreased, partly explaining the declining possibility of implant placement unrelated to the maxillary sinus towards the anterior aspect. The gradual reduction in residual bone height from posterior to anterior observed in this study contrasts with the findings of Manzanera *et al*. where the bone height at 3 mm from the distal aspect of the second maxillary molar was higher than at 6 mm in cases of severe tuberosity resorption (21). This discrepancy could be attributed to including patients with retained second molars in Manzanera’s study. The presence of the second molar may have contributed to maintaining a higher bone height at 3 mm from its distal aspect compared to 6 mm, or, in other words, the loss of the second molar not only reduced bone height directly at its site but also at distant locations.

The possibility of placing pterygoid implants without maxillary sinus intrusion significantly decreased from position A (85.8%) to position E (43.3%). The success rate of pterygoid implant placement in the study by Zhang *et al*. (18) was 67%, lower than positions A (85.8%), B (81.7%), and C (70.8%), and higher than positions D (58.3%) and E (43.3%) in our study. Meanwhile, the pterygoid implant neck positions reported by Zhang *et al*. (18) were 12.91±2.07 mm distant from the most inferior posterior point of the maxillary tuberosity, corresponding to positions D and E in our study. This difference may stem from variations in the study population. At the same time, Zhang *et al*.’s sample (18) included dentate and edentulous patients, while our sample consisted solely of completely edentulous patients with severe bone resorption. In the study by Sun *et al*. (19) the pterygoid implant neck position was approximately 10 mm from the pterygomaxillary joint, corresponding to position D in our study. However, the possibility of placing pterygoid implants without maxillary sinus involvement only reached 9.4%, compared to 58.3% in our study. This could be attributed to the difference in the angulation in the anterior-posterior direction relative to the Frankfort horizontal plane; Sun *et al*. (19) reported a fixed angle at 45o, whereas the angulation in our study at position D ranged from 25.5o to 58.5o. As the pterygoid implant neck is placed more posteriorly, the likelihood of maxillary sinus penetration decreases but potentially poses challenges in patient oral hygiene maintenance (18). However, in this study, the rate of maxillary sinus intrusion at positions A and B was equivalent, suggesting that a position approximately 8 mm from the most posterior point of the tuberosity may be the better choice compared with the 6 mm position for an optimal entrance location for pterygoid implants.

Similar to the study conducted by Uchida *et al*. (17) this research findings demonstrate that the angle of the pterygoid implant relative to the Frankfort horizontal plane in the sagittal plane varied from 68±6.9o to 32.7±8.5o when the implant neck was positioned from posterior to anterior of the tuberosity (17). This explains why, in clinical practice, some authors recommend placing pterygoid implants with a 70o angulation (13), while others suggest a 45o angulation (12,14). This variation in angulation depends on the implant neck position. At position A, the sagittal angulation was 68±6.9o, close to the angles reported in the studies by Rodriguez *et al*. (74.19o) and Uchida *et al*. (75.1o) (15-17). Meanwhile, the angulation at points C (50.1±7.9o) and D (40.7±8.0o) was similar to that reported in the study by Zhang *et al*. (45.08±2.56o) (18). In the study by Zhang *et al*., the implant neck positions correspond to positions D and E in our study; however, the sagittal angulation in our research, 40.7±8.0o, and 32.7±8.5o, respectively, was smaller than that reported by Zhang *et al*. (18). This may be because, in our study, all patients were utterly edentulous, potentially leading to more severe maxillary sinus pneumatization, resulting in minor sagittal angulation of the implant.

The process of implanting pterygoid implants in the management of total maxillary edentulism requires great care to avoid impacting vital anatomical structures, by tilting the implant posteriorly. However, the tilted angulation should not exceed 45o to fulfill a need from the biomechanical aspects and prosthetic fabrication procedures (22). Furthermore, anterior positioning of the pterygoid implant neck is recommended to facilitate patient oral hygiene maintenance (18). To satisfy the above criteria, our research shows that the neck of the implant should be placed at a distance of 8, 10, or 12 mm from the furthest point of the tuberosity. The average distance of the optimal implant neck to the furthest point of the tuberosity is 9.79±2.19 mm, with the angulation in the anteroposterior and medial-lateral direction relative to the Frankfort horizontal plane being 51.82±5.57º and 74.15±16.53º, respectively. This suggests that during virtual implant placement in software, clinicians should start with the neck of the implant about 10 mm from the furthest point of the tuberosity. Moreover, the furthest point of the tuberosity (M point) can be determined in the operating procedure after flap creation and dissection, allowing the surgeon to directly identify the entry point for the drill in the clinical corresponding to the plan set on CBCT.

Pterygoid implantations fluctuating in length from 15 to 18 mm were predominantly utilized, exceeding the recommended size in Zhang *et al*.’s study (18), likely attributed to our method of achieving optimal initial stability by minimal penetration into the pterygoid fossa. Notably, Sun *et al*.’s (19) virtual pterygoid implants were also positioned within the pterygoid fossa similarly to our study, albeit with a recommended length of 16 mm, lower than in our investigation, possibly due to discrepancies in implant neck positioning and angulation in the sagittal and buccal-lingual directions.

Guided surgery techniques involving both static and dynamic navigated systems has dramatically enhanced implant placement accuracy and predictability for pterygoid implant placement. Static guided surgery utilizes prefabricated guides derived from preoperative imaging studies such as CBCT scans to adhere closely to planned trajectories, shorten surgical duration, and minimize surgical times. However, static guides may encounter limitations when dealing with unexpected anatomical variations and require a steep learning curve for accurate design and fabrication (23).

Dynamic navigation guided surgery offers real-time feedback, which facilitates intraoperative adjustments and adaptability to anatomical challenges (24). While its advantages outweigh its drawbacks, navigated surgery requires extensive training in order to utilize real-time data effectively (25). A comparison between static and dynamic navigation guided surgery for pterygoid implants shows both methods enhance implant placement accuracy relative to freehand techniques; however, navigated surgery’s dynamic feedback mechanisms provide superior adaptability in complex anatomical regions (26,27).

Our research offers significant insights into the optimal positioning, localization, length and angulation of pterygoid implants - key elements in effective planning. Further investigations should compare freehand techniques performed by highly experienced surgeons with guided surgeries conducted by less experienced practitioners - to assess relative efficacy and adaptability between guided versus freehand approaches across clinical scenarios, ultimately informing best practices and training protocols in implantology (27).

This study utilizes CBCT imaging to describe in great depth the maxillary tuberosity and pterygoid area in fully edentulous individuals using CBCT imaging, providing a more in-depth picture. However, given it is an *in vitro* study it would be essential to conduct long-term clinical research to validate and prove these findings as well as ensure their applicability in improving clinical outcomes for those suffering severe maxillary bone resorption (8).

## Conclusions

The quantity of bone on the maxillary tuberosity and the posterior wall of the maxillary sinus determines the pterygoid implant’s location, angulation, and length. In completely edentulous patients, pterygoid implants should be positioned approximately 10 mm away from the farthest point of the maxillary tuberosity at an angle of roughly 50 degrees anteroposterior and 75 degrees buccal-lingual. However, determining the optimal position and orientation of the implant should be based on the individual CBCT data of each patient. An implant length of 18mm is the most commonly used; however, in some cases, implants with lengths of 20 to 22 mm may be necessary to achieve bicortical anchorage.

## Figures and Tables

**Table 1:** Possibility to place implants without sinus intrusion at various positions (Chi-Square Test).

Positions	A	B	C	D	E	
Right (n,%)	54 (90%)	50 (83.3%)	43 (71,7%)	39 (65%)	30 (50%)	P<0.05
Left (n, %)	50 (83.3%)	48 (80%)	42 (70%)	31 (51.7%)	22 (36.7%)	
Total	104 (85.8%)	98 (81.7%)	85 (70.8%)	70 (58.3%)	52 (43.3%)	

**Table 2:** Angle of the implant relative to the Frankfort horizontal plane in the anterior-posterior and buccal-lingual directions at various positions.

Positions(^o^)	A (^o^)	B (^o^)	C (^o^)	D (^o^)	E (^o^)	P value
Antero-Posterior Angle (^o^)	68±6.9	58.4±8.5	50.1±7.9	40.7±8.0	32.7±8.5	P<0.05
Buccal-Lingual angle (^o^)	78.2±5.4	76.3±5.7	74.2±6.7	70.4±8.5	67.6±10.8	P<0.05

**Table 3:** Distribution of pterygoid implants length used at various positions.

Implant Length (mm)	A	B	C	D	E
10-13 (n,%)	16 (15.4)	7 (7.1)	4 (4.7)	0 (0)	0 (0)
15 (n,%)	45 (43.3)	37 (37.8)	20 (23.5)	11 (15.7)	1 (1.9)
18 (n,%)	33 (31.7)	38 (38.8)	43 (50.6)	34 (48.6)	24 (46.2)
20-22 (n,%)	10 (9,6)	18 (18.8)	18 (21.2)	25 (35.7)	27 (51.9)

**Table 4:** Optimal position, angulation, and length of pterygoid implants.

Position (n,%)	A (11, 10.6)	B (27, 26)	C (36, 34.6)	D (22, 21.2)	E (8, 7.7)	Mean
Antero-Posterior Angle (^o^)	58±8.3	53.14±5.44	51.2±4.87	49.2±2.78	48.81±2.61	51.82±5.57
Buccal-Lingual angle (^o^)	76.86±5.27	73.85±5.92	75.33±6.68	73.22±7.29	68.68±4.5	74.15±16.53
Implant length	13 (11.5-15)	15 (15-15)	18 (15-18)	18 (18-20)	21 (20-22)	18 (15-18)

## Data Availability

The datasets used and/or analyzed during the current study are available from the corresponding author.
